# Hyperspectral imaging-based classification of rice leaf blast severity over multiple growth stages

**DOI:** 10.1186/s13007-022-00955-2

**Published:** 2022-11-19

**Authors:** Guosheng Zhang, Tongyu Xu, Youwen Tian

**Affiliations:** Shenyang Agricultural University, Shenyang, China

**Keywords:** Rice leaf blast, Hyperspectral imaging, Spectral reflectance ratio, Multiple growth stages, Generalization

## Abstract

**Background:**

Rice blast, which is prevalent worldwide, represents a serious threat to harvested crop yield and quality. Hyperspectral imaging, an emerging technology used in plant disease research, is a stable, repeatable method for disease grading. Current methods for assessing disease severity have mostly focused on individual growth stages rather than multiple ones. In this study, the spectral reflectance ratio (SRR) of whole leaves were calculated, the sensitive wave bands were selected using the successive projections algorithm (SPA) and the support vector machine (SVM) models were constructed to assess rice leaf blast severity over multiple growth stages.

**Results:**

The average accuracy, micro F1 values, and macro F1 values of the full-spectrum-based SVM model were respectively 94.75%, 0.869, and 0.883 in 2019; 92.92%, 0.823, and 0.808 in 2021; and 88.09%, 0.702, and 0.757 under the 2019–2021 combined model. The SRR–SVM model could be used to evaluate rice leaf blast disease during multiple growth stages and had good generalizability.

**Conclusions:**

The proposed SRR data analysis method is able to eliminate differences among individuals to some extent, thus allowing for its application to assess rice leaf blast severity over multiple growth stages. Our approach, which can supplement single-stage disease-degree classification, provides a possible direction for future research on the assessment of plant disease severity during multiple growth stages.

## Background

Rice, a crop cultivated worldwide, accounts for approximately one-quarter of the total crop planting area in China and one-third of the grain yield [1]. Rice blast caused by *Magnaporthe grisea* occurs in almost every rice-growing country and region [2] and negatively impacts potential crop yield and quality. Epidemics due to this fungus typically result in 10–20% reductions in production, with greater than 40% reductions observed in severe cases [3]. Since the 1990s, the annual area of rice blast occurrence in China has averaged at least 38,000 km^2^, and annual losses have been up to several hundred million kilograms [4]. To date, one of the most widely used methods for controlling rice blast is spraying with fungicide [5, 6]. Under field conditions, however, most disease is nonhomogeneously distributed [7]. Uniform spraying requires an excessive amount of agrochemicals, resulting in increased costs, environmental pollution, and fungal resistance [8, 9]. Consequently, the accurate evaluation of rice blast severity is an economically important aspect of precision agriculture.

During pathogenesis, *M. grisea* undergoes a complex sequence of developmental and metabolic events [10]. Four types of lesions have been found under field conditions: acute, chronic, white spot, and brown spot forms [3]. Current disease scouting and phenotyping techniques rely on human observations and visual ratings [11–13]. Visual ratings, which are dependent on rater ability and reliability, may be prone to human error, subjectivity, and inter/intra-rater variability [14–17]. To overcome these shortcomings, remote sensing techniques have been introduced to provide an easily available, permanent record of disease intensity without the problems associated with human rating [18].

Hyperspectral imaging is an emerging means of assessing plant vitality, stress parameters, nutrition status, and disease [19]. This technique produces digital measurements that can easily be shared and quickly analyzed using semi-automated procedures in a repeatable and objective manner [20]. In addition, hyperspectral imaging can be used to measure reflectance in visible, near-infrared, and even short-wave infrared ranges, thereby providing more abundant information [13, 21]. Hyperspectral imaging has been widely used to assess plant disease severity. For instance, Thomas et al. [22] used hyperspectral imaging to investigate the powdery mildew resistance of six barley cultivars up to 30 days after inoculation. Oerke et al. [23] quantified *Cercospora beticola* sporulation in sugar beet leaves using hyperspectral microscopy to assess the resistance of different genotypes. Jiang et al. [24] estimated the severity of mangrove diseases carried by herbivorous insects using a random forest model with the optimal R^2^ of 0.752. Gui et al. [25] established a combined convolutional neural network and support vector machine method to grade soybean mosaic disease and achieved an accuracy as high as 94.17%. Coops et al. [26] detected three levels of Dothistroma needle blight infection with an accuracy of over 70% using airborne hyperspectral remote sensing imagery.

Hyperspectral imaging has been effectively used in previous studies and has greatly promoted plant disease research. To our knowledge, however, no classification method exists to assess rice leaf blast severity over multiple growth stages. Fungal plant pathogens affect almost all relevant crops during different stages of development [27]. To evaluate plant diseases at different growth stages, several classification models are currently required to encompass different time periods, a time-consuming practice. The development of an efficient method to assess disease severity is therefore important.

In this study, hyperspectral images of rice leaves were obtained using a ground-based hyperspectral imaging system in 2019 and 2021 period. The average spectral reflectance of whole leaves and healthy leaf tissues were extracted with ENVI 5.6. A spectral reflectance ratio (SRR) data analysis method was used for data processing and the successive projections algorithm (SPA) was used to select sensitive wave bands. Full-spectrum-based SVM models and SPA-SVM models were constructed to assess rice leaf blast severity over multiple growth stages, and the generalizability of the model was evaluated.

## Materials and methods

### Plant materials

All experiments were conducted in Liaoning Province, China, using rice blast-susceptible Mongolian rice. All samples were directly obtained from a naturally infected field. The first portion of the study took place in 2019 in Shenyang (123°63′ E, 42°01′ N). Experimental plants were sown on May 23. The row space of rice plants was 0.2 m, and the line space of those was about 0.35 m. Urea–potassium sulfate–superphosphate fertilizer was applied basally at a rate of 270, 80, and 130 kg/ha, respectively, with additional urea supplied at a rate of 50 kg/ha at the tillering stage. To eliminate the influence of insect pests, 5 g of chlorpyrifos 74% wettable powder (Shanghai Nongle Agricultural Chemical Co., Shanghai, China) was mixed with 10 kg of water to form a solution that was applied monthly with a T20 UAV sprayer (SZ DJI Technology Co., Shenzhen, China). The second portion of the study was conducted in 2021 at Haicheng (122°73′ E, 40°98′ N). Experimental plants were sown on May 25. All other management practices were the same as in 2019. Twelve diseased rice plants and two healthy ones were randomly selected from the field at three different growth stages. The rice plants were placed in a barrel (42 cm diameter and 50 cm deep). Soil and water in the field were added in the barrel to maintain the state of rice plants. The barrels were transported to a hyperspectral imaging room, and hyperspectral images of rice leaves were acquired the following day. All samples were divided into a training set and a testing set at a ratio of 7:3. Specific descriptions of samples are provided in Table 1.

**Table 1:** Collection date and numbers of samples in this study

Year	Growth stage	Collection date	Samples	Quantity
2019	Jointing stage	July 8 and	Level 0	15
		15-Jul	Level 1	16
			Level 2	36
			Level 3	32
			Level 4	21
			Total	120
	Booting stage	July 25 and	Level 0	18
		2-Aug	Level 1	19
			Level 2	32
			Level 3	18
			Level 4	18
			Total	105
	Heading stage	10-Aug	Level 0	19
			Level 1	11
			Level 2	28
			Level 3	21
			Level 4	26
			Total	105
2021	Jointing stage	13-Jul	Level 0	10
			Level 1	20
			Level 2	15
			Level 3	9
			Level 4	1^*^
			Total	55
	Booting stage	27-Jul	Level 0	18
			Level 1	49
			Level 2	14
			Level 3	28
			Level 4	18
			Total	127
	Heading stage	12-Aug	Level 0	15
			Level 1	45
			Level 2	20
			Level 3	34
			Level 4	23
			Total	137

^*^There was only one sample of level 4, so it acted as the training set and the testing set simultaneously

### Hyperspectral imaging

**Fig. 1:**
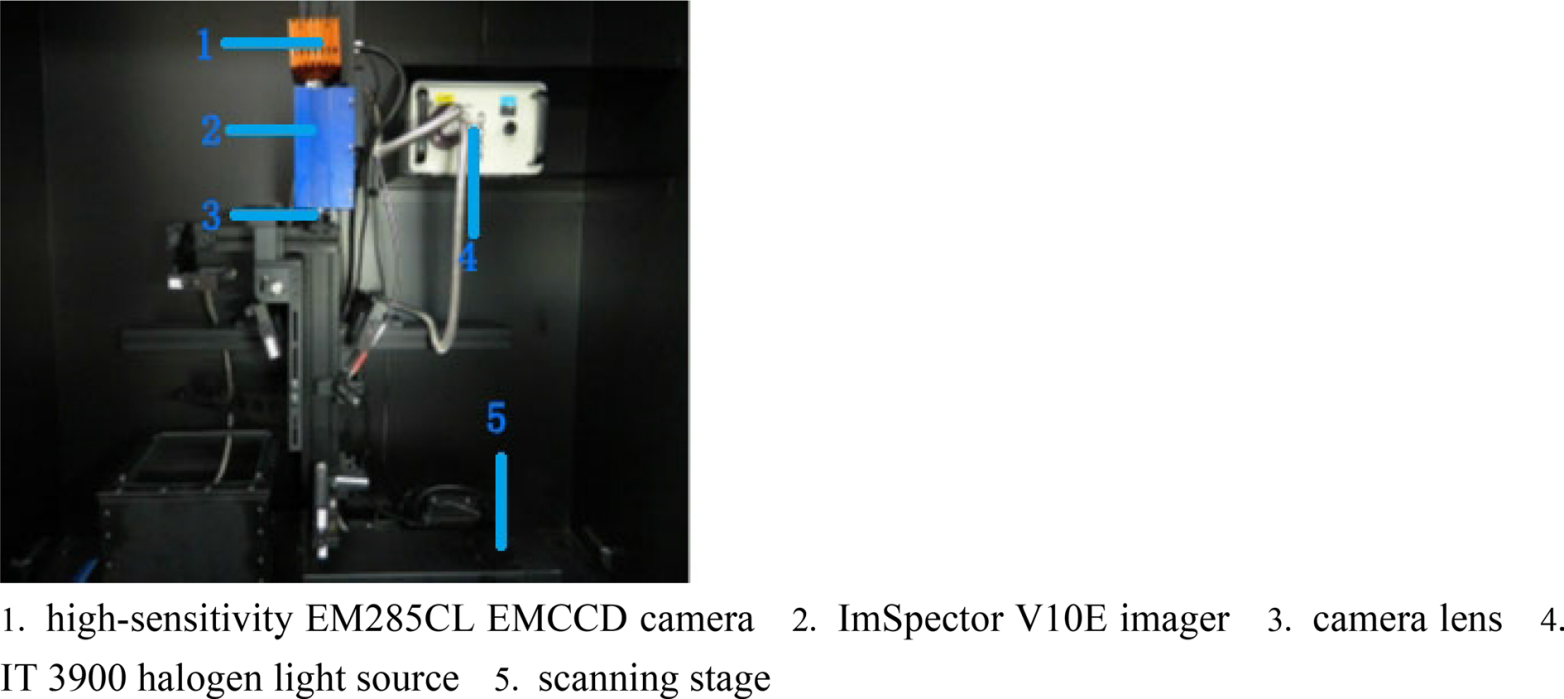
Hyperspectral imaging system used in this study

The imaging system (Fig. 1) consisted of a high-sensitivity EM285CL EMCCD camera (Raptor Photonics, Antrim, Northern Ireland), a stand-mounted ImSpector V10E imager (Spectral Imaging, Oulu, Finland), a horizontally movable scanning stage, a desktop computer with Spectral-Image software (Isuzu Optics, Hsinchu, China) for controlling the imager and scanning stage during image collection, and an IT 3900 halogen light source (Ocean Optics, Dunedin, FL, USA) to provide stable illumination. The ImSpector V10E imager collected 472 wavebands over a spectral range of 400–1000 nm. The angle of the left and right linear emitters was adjusted to a vertical orientation of 45° to enable the emitted light rays to converge on a coincident line just below the camera lens. The objective lens of the camera was set at an aperture of f/1.4. The distance between the camera lens and the scanning stage was set to 300 mm, and the focus was manually adjusted to guarantee image definition. The exposure time was manually adjusted according to the lighting environment to ensure sufficient incident radiation intensity. The speed of the scanning stage was set to 1.2 mm/s, with the aspect ratio set to the default. Leaves were carefully removed from each rice stem, placed flat on the stage, and gently affixed with double-sided adhesive. Five columns of rice leaves were placed parallel to one another on the scanning stage while the camera ran at every turn. Great care was taken to avoid exerting any pressure on the leaves. Rice leaves longer than 400 mm, the maximum sliding distance of the scanning stage, were cut into two or more sections while preserving the integrity of the diseased area. Images were captured using Spectra-Image software, and the hyperspectral data cubes were saved onto an external hard drive.

The original hyperspectral images were corrected each time for dark current and uneven light intensity distribution before further processing [28]. First, a white board with high reflectance was scanned as a 100% standard. The value of Max DN was adjusted to 3600, which was 80% of the maximum value, and the bright field of the white board was recorded. After covering the lens cap, the dark field of the white board was measured. The white board was then removed. Next, the samples were situated directly below the camera on the scanning stage, with the exposure time adjusted to keep the value of Max DN at 3600 with the other parameters unchanged. After covering the lens cap, the dark field of the sample was recorded. The corrected sample image was calculated as follows:

1 $$R=\frac{{R}_{s}-{R}_{sd}}{{R}_{bw}-{R}_{bd}}$$

where *R* is the corrected sample image, ${R}_{s}$ is the original hyperspectral image of the sample, ${R}_{sd}$ is the hyperspectral image of the dark field of the sample, ${R}_{bw}$ is the hyperspectral image of the bright field of the white board, and ${R}_{bd}$ is the hyperspectral image of the dark field of the white board.

### Classes of disease severity

Rice leaves were manually traced using the ROI tool in ENVI 5.6 (ITT Visual Information Solutions, Boulder, CO, USA). The area of the rice leaf was selected as a region of interest (ROI), with the number of pixels contained therein counted automatically and recorded as N1. The number of pixels in diseased areas was calculated in the same way and recorded as N2. The degree of rice blast on a leaf was calculated as the percentage of the leaf covered by lesions relative to the whole leaf area, that is, the value of (N2 / N1) × 100%. Disease severity was then classified according to [29] into six levels as follows: 0, no visible lesions; 1, up to 1% of the leaf showing rice blast symptoms; 2, 1% to 5% showing symptoms; 3, 5% to 10% showing symptoms; 4, 10% to 50% showing symptoms; and 5, over 50% showing symptoms. Because samples assigned to level 5 were observed only under extremely severe disease conditions, only five classes of samples (levels 0 to 4) were discriminated in this study.

### Analysis of the hyperspectral dataset

HSI Analyzer (Isuzu Optics, Hsinchu, China) was used to normalize the hyperspectral images against known values of the white reference standard. The whole rice leaf was manually traced using the ROI tool and selected as a ROI. The average spectral reflectance of the ROI was then extracted and saved. Depending on the size of the leaf, three or more rectangular areas of the same size were selected. All of the rectangular areas were distributed in different positions of the leaf. The selected areas were treated as a single ROI, and the average spectral reflectance of the ROI was extracted and saved. The spectral reflectance data were analyzed using a SRR data analysis method proposed by Zhang et al. [30] as follows:

2 $$SRR=\frac{{R}_{h}}{{R}_{w}}$$

where R_w_ is the average spectral reflectance of the whole leaf, and R_h_ represents the average spectral reflectance of healthy parts of the same leaf.

The acquired spectral reflectance consisted of two parts: true value and noise. Equation (2) can thus be expressed as:

3 $$SRR=\frac{{R}_{H}+{R}_{NH}}{{R}_{W}+{R}_{NW}}$$

where R_H_ is the true value of R_h_, R_NH_ is the noise of R_h_, R_W_ is the true value of R_w_, and R_NW_ is the noise of R_w_.

Spectral noise has two components: air absorption and equipment noise. Air absorption is affected by the distance between pixels and the lens, whereas equipment noise is influenced by voltage. The width of rice leaves is only approximately 1 cm, which is roughly 1/30 of the object distance. As a result, the distribution of pixels is irrelevant when calculating pixel–lens distances. For the whole leaf, air absorption can be considered to be constant. In a single imaging run, the noise generated by the hyperspectral imaging system remains unchanged. Equipment noise also stays the same for the whole leaf. Overall, R_NH_ is equal to R_NW_ for a single leaf, and both variables can be assigned as R_N_. Equation (3) can thus be written as:

4 $$SRR=\frac{{R}_{H}+{R}_{N}}{{R}_{W}+{R}_{N}}=\frac{{R}_{H}}{{R}_{W}}+\frac{{R}_{N}({R}_{W}-{R}_{H})}{{R}_{W}({R}_{W}+{R}_{N})}$$

Compared with R_W_, R_N_ is extremely small after processing of the above-mentioned white and dark references. R_N_/R_W_ can thus be regarded as infinitesimal; the absolute value of (R_W_-R_H_) / (R_W_ + R_N_) is smaller than 1, and their product is still infinitesimal. Equation (4) can be simplified as:

5 $$SRR=\frac{{R}_{H}}{{R}_{W}}$$

As can be seen from (5), the value of SRR only depends on the true value of hyperspectral reflectance, thereby demonstrating its capacity in noise resistance. In addition, the value of SRR does not depend entirely on spectral reflectance, thus eliminating differences among individuals to some extent. The value of SRR therefore indicates the change rate of spectral reflectance of a rice leaf after infection by *M. grisea*: in other words, the degree of rice leaf blast severity.

### Classification model construction and evaluation

The SRR dataset was classified into different degrees of disease severity by a non-linear SVM [31]. The applied SVM used the radial basis function as the kernel function to determine non-linear discriminant functions. In this study, randomly selected samples were chosen as the training set, and the remaining samples were assigned to the testing set. To build the optimal SVM model, the penalty parameter of the error term *C* and the kernel parameter *g* were optimized using a fivefold grid-search optimization [32]. The range of *C* was set as 10^ N^ (− 10 ≤ *N* ≤ 10, with a step size of 0.1), and that of *g* was 10^ M^ (− 15 ≤ *M* ≤ 5, with a step size of 0.1). The best penalty parameters were determined according to the highest cross-validation accuracy of the training set. The classification performances of the SVM models were evaluated using the average accuracy, micro F1 value, and macro F1 value of the testing set [33]. LIBSVM 3.23 [34] was used to construct models and is available at https://www.csie.ntu.edu.tw/~cjlin/libsvm/index.html. Data analysis and model construction were conducted in MATLAB 2016b (MathWorks, Natick, MA, USA).

## Results

### Spectral reflectance signatures of leaves of different levels

**Fig. 2:**
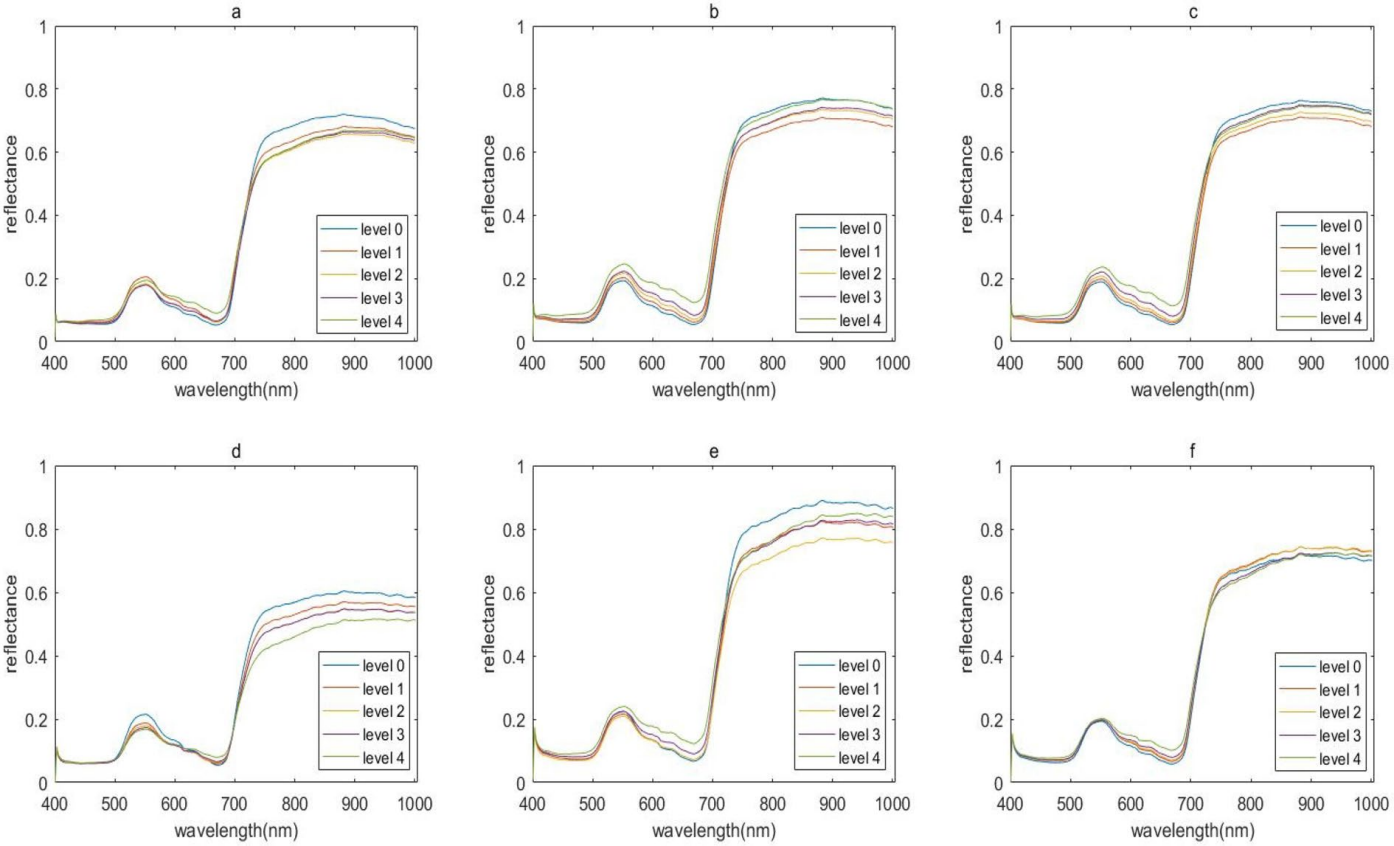
Raw spectral reflectance of leaf samples exhibiting different levels of disease severity at three growth stages. **a**–**c** Jointing (**a**), booting (**b**), and heading (**c**) stages in 2019. **d**–**f** Jointing (**d**), booting (**e**), and heading (**f**) stages in 2021

There existed some differences of spectral reflectance among leaves of 5 levels in 3 growth stages, but not evident (Fig. 2). In the visible region, the average spectral reflectance of infected leaves was higher than the healthy ones, the value of spectral reflectance increased with disease severity deepening; while in the near-infrared region, the spectral reflectance of diseased leaves was lower than that of healthy ones, the value of spectral reflectance increased with the severity of disease deepening, and the curves among samples of different grades tended to be parallel. Different rice leaf blast severity had a diverse effect on both visible and near-infrared bands.

**Fig. 3:**
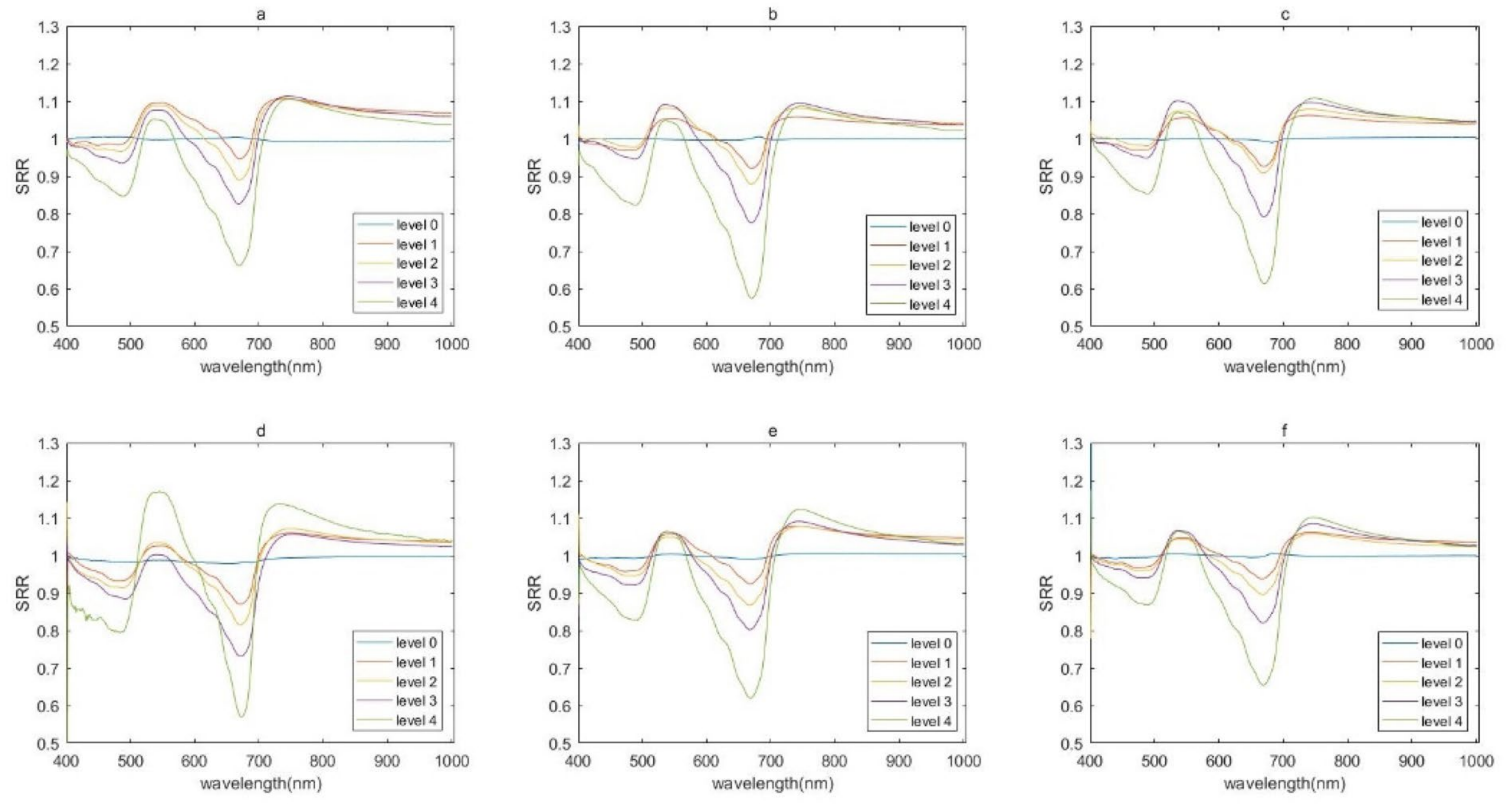
SRR of leaf samples exhibiting different levels of disease severity at three growth stages. **a**–**c** Jointing (**a**), booting (**b**), and heading (**c**) stages in 2019. **d**–**f** Jointing (**d**), booting (**e**), and heading (**f**) stages in 2021

### Spectral characterization of SRR

Differences in SRR were significant among samples at different levels of disease severity (Fig. 3). In healthy leaves, the SRR curve was approximately a straight line, with a value of 1 observed across the entire spectral region. In diseased leaves, the most distinct differences in the visible region were centered at approximately 491 nm and 667 nm regardless of growth stage or year. The value of SRR in the vicinity of these troughs decreased as disease severity increased. In the near-infrared region, the SRR curves tended to coincide, with no obvious differences among leaves at different disease levels. SRR curve profiles were similar at all three growth stages in both years, whereas the SRR values were unequal.

### Assessment of rice leaf blast severity over multiple growth stages

The full-spectrum-based SVM models performed well for assessing rice leaf blast severity over multiple growth stages, as well as the SPA-SVM models (Table 2). Average accuracies in both years exceeded 90%, and micro and macro F1 scores were around 0.8. All misclassifications occurred between adjacent disease levels (Table 3). The performances of the full-spectrum-based SVM models were a little better than the SPA-SVM models (Table. 3).

**Table 2:** Model parameters and performances for SVM models throughout multiple growth stages

Year	Bands	Sample	Trainning set	Testing set	Model	Average accuracy	Micro	Macro
					Parameters		F1 score	F1 score
2019	Full	Level 0	36	16	C = 84.4485	\	\	\
		Level 1	32	14	G = 0.0063457	\	\	\
		Level 2	67	29		\	\	\
		Level 3	50	21		\	\	\
		Level 4	46	19		\	\	\
		Total	231	99		94.75%	0.869	0.883
	491 nm	Level 0	36	16	C = 36.7583	\	\	\
	671 nm^*^	Level 1	32	14	G = 8	\	\	\
		Level 2	67	29		\	\	\
		Level 3	50	21		\	\	\
		Level 4	46	19		\	\	\
		Total	231	99		92.73%	0.818	0.833
	491 nm	Level 0	36	16	C = 51.9842	\	\	\
	668 nm^*^	Level 1	32	14	G = 3.7321	\	\	\
		Level 2	67	29		\	\	\
		Level 3	50	21		\	\	\
		Level 4	46	19		\	\	\
		Total	231	99		92.32%	0.808	0.816
2021	All	Level 0	30	13	C = 1024	\	\	\
		Level 1	80	34	G = 0.0019531	\	\	\
		Level 2	34	15		\	\	\
		Level 3	50	21		\	\	\
		Level 4	29	13		\	\	\
		Total	223	96		92.92%	0.823	0.808
	491 nm	Level 0	30	13	C = 13.9288	\	\	\
	671 nm	Level 1	80	34	G = 3.0314	\	\	\
		Level 2	34	15		\	\	\
		Level 3	50	21		\	\	\
		Level 4	29	13		\	\	\
		Total	223	96		92.08%	0.802	0.713
	491 nm	Level 0	30	13	C = 238.8564	\	\	\
	668 nm	Level 1	80	34	G = 0.35355	\	\	\
		Level 2	34	15		\	\	\
		Level 3	50	21		\	\	\
		Level 4	29	13		\	\	\
		Total	223	96		92.5%	0.813	0.793

^*^491 and 671 nm were selected sensitive wave bands for SRR data acquired in 2019 using SPA

^*^491 and 668 nm were selected sensitive wave bands for SRR data acquired in 2021 using SPA

**Table 3:** Predictions of SVM models throughout multiple growth stages

Year	Bands	Sample	Level 0	Level 1	Level 2	Level 3	Level 4	True
2019	All	Level 0	16	0	0	0	0	16
		Level 1	0	11	3	0	0	14
		Level 2	0	0	27	2	0	29
		Level 3	0	0	5	15	1	21
		Level 4	0	0	0	2	17	19
		Prediction	16	11	35	19	18	99
	491 nm	Level 0	15	1	0	0	0	16
	671 nm	Level 1	1	11	2	0	0	14
		Level 2	0	1	24	4	0	29
		Level 3	0	0	5	16	0	21
		Level 4	0	0	0	4	15	19
		Prediction	16	13	31	24	15	99
	491 nm	Level 0	15	1	0	0	0	16
	668 nm	Level 1	3	8	3	0	0	14
		Level 2	0	0	24	5	0	29
		Level 3	0	0	5	16	0	21
		Level 4	0	0	0	2	17	19
		Prediction	18	9	32	23	17	99
2021	All	Level 0	13	0	0	0	0	13
		Level 1	0	34	0	0	0	34
		Level 2	0	10	3	2	0	15
		Level 3	0	0	1	19	1	21
		Level 4	0	0	0	3	10	13
		Prediction	13	44	4	24	11	96
	491 nm	Level 0	13	0	0	0	0	13
	671 nm	Level 1	2	32	0	0	0	34
		Level 2	0	9	0	6	0	15
		Level 3	0	0	0	21	0	21
		Level 4	0	0	0	2	11	13
		Prediction	15	41	0	29	11	96
	491 nm	Level 0	12	1	0	0	0	13
	668 nm	Level 1	1	33	0	0	0	34
		Level 2	0	8	2	5	0	15
		Level 3	0	0	1	20	0	21
		Level 4	0	0	0	2	11	13
		Prediction	13	42	3	27	11	96

### Generalizability of the classification model

Generalizability of the SRR–SVM model was evaluated by analyzing the performance of a 2019–2021 combined model. The training set comprised all samples acquired in 2019, and the testing set consisted of those collected in 2021. The 2019–2021 combined model performed well, although its performance was slightly worse than models covering a single year (Table 4). Most misclassifications occurred between adjacent disease levels (Table 5). Samples tended to be misclassified as level 2. The The performances of the full-spectrum-based SVM model was slightly worse than the SPA-SVM models, but not obvious.

**Table 4:** Model parameters and performances for the 2019–2021 combined model

Bands	Sample	Trainning	Testing	Model	Average	Micro	Macro
		Set	Set	Parameters	Accuracy	F1 score	F1 score
All	Level 0	52	43	C = 119.4282	\	\	\
	Level 1	46	114	G = 0.011049	\	\	\
	Level 2	96	49		\	\	\
	Level 3	71	71		\	\	\
	Level 4	65	42		\	\	\
	Total	330	319		88.09%	0.702	0.757
491 nm	Level 0	52	43	C = 315.173	\	\	\
668 nm	Level 1	46	114	G = 16	\	\	\
671 nm^*^	Level 2	96	49		\	\	\
	Level 3	71	71		\	\	\
	Level 4	65	42		\	\	\
	Total	330	319		89.47%	0.709	0.759

^*^ 491, 668 and 671 nm were the combination of selected sensitive wave bands of SRR data acquired in 2019 and 2021

**Table 5:** Predictions of the 2019–2021 combined model

Wavelength	Sample	Level 0	Level 1	Level 2	Level 3	Level 4	True
Full	Level 0	41	1	1	0	0	43
	Level 1	0	67	43	4	0	114
	Level 2	1	4	35	9	0	49
	Level 3	0	2	19	43	7	71
	Level 4	0	0	0	4	38	42
	Prediction	42	74	98	60	45	319
491 nm	Level 0	40	3	0	0	0	43
668 nm	Level 1	7	79	25	3	0	114
671 nm	Level 2	0	7	29	13	0	49
	Level 3	0	3	16	51	1	71
	Level 4	0	0	0	6	36	42
	Prediction	47	92	70	73	37	319

## Discussion

In theory, the SRR curve for healthy leaves should have been a straight line of value 1, as no lesions were present; in fact, the curve had subtle fluctuations, possibly the result of the different proportion of veins and mesophyll between the whole leaf and our selected ROI. In general, more water, less solid matter, and less air are present in veins [35]. This unbalanced distribution of substances between veins and mesophyll may cause the average reflectance to differ between the whole leaf and the ROI, resulting in an SRR not exactly equal to 1.

In regard to diseased leaves, the most notable differences were observed at approximately 491 nm and 667 nm, which correspond to carotenoid and chlorophyll absorption bands. This result indicates that infection by rice leaf blast increased the spectral reflectance and reduced the carotenoid and chlorophyll contents of the studied leaf area [36–38]. The profiles of leaves at different disease levels tended to coincide in the near-infrared region, which indicates that disease severity was not a major contributor to the shape of the curve in this region—unlike the situation in the visible region. Detecting and assessing disease severity in the near-infrared region may therefore be difficult [5, 39–42].

The pure SVM classifier exploits the characteristics of hyperspectral imaging via the kernel function by combining spectral with spatial features [39]. Average accuracy reflects the average per-class effectiveness of a classifier. Micro and macro F1 values indicate the relationship between a dataset’s positive labels and those given by a classifier based respectively on sums of per-text decisions or on a per-class average [33]. In our study, most misclassifications occurred between samples at adjacent disease levels. This result may have been due to two different phenomena. First, a single leaf may have contained various forms of lesions whose areas differed from one another. Despite the identical disease level, the spectral reflectance would thus have fluctuated. Second, biological heterogeneity may have contributed to the inaccuracy of classification [43]. Different leaves may have had unequal levels of vitality; although they had the same type of lesions with similar areas, their spectral reflectance may still have been different.

Zhang et al. [39] also established a disease monitoring model for wheat Fusarium head blight covering more than one stage, but their samples were all collected within one week (3 May 2018 [at the late flowering stage] and 9 May 2018 [at the early filling stage]). In contrast, our sample collection spanned more than a month and included the period most conducive to rice leaf blast occurrence under field conditions. Zhang et al. [30] also used this method to classify rice leaf blast severity; however, they focused on a single growth stage, not multiple growth stages as in our study.

Although the performance of our method is good, some problems remain. First, the processing of hyperspectral images is still too labor intensive, hindering the inspection of large numbers of samples. Second, the experiment was conducted under a controlled environment, and the results cannot be easily extended to field conditions. In the future, we hope to resolve these two issues.

## Conclusions

In this study, a SRR data analysis method was applied and full-spectrum-based SVM models were constructed to assess rice leaf blast severity over multiple growth stages. The degree of rice leaf blast severity based on the area covered by lesions relative to the whole leaf area was determined, and, from the perspective of spectral reflectance, the SRR value was found to reflect the disease level. Our results should provide a possible direction for assessing plant disease severity over multiple growth stages.

## Data Availability

The datasets used and/or analysed during the current study available from the corresponding author on reasonable request.
